# A Computational Model for Inferring QTL Control Networks Underlying Developmental Covariation

**DOI:** 10.3389/fpls.2019.01557

**Published:** 2019-12-18

**Authors:** Libo Jiang, Hexin Shi, Mengmeng Sang, Chenfei Zheng, Yige Cao, Xuli Zhu, Xiaokang Zhuo, Tangren Cheng, Qixiang Zhang, Rongling Wu, Lidan Sun

**Affiliations:** ^1^Beijing Advanced Innovation Center for Tree Breeding by Molecular Design, Center for Computational Biology, College of Biological Sciences and Biotechnology, Beijing Forestry University, Beijing, China; ^2^Beijing Advanced Innovation Center for Tree Breeding by Molecular Design, Beijing Key Laboratory of Ornamental Plants Germplasm Innovation & Molecular Breeding, National Engineering Research Center for Floriculture, Beijing Laboratory of Urban and Rural Ecological Environment, College of Landscape Architecture, Beijing Forestry University, Beijing, China; ^3^Center for Statistical Genetics, The Pennsylvania State University, Hershey, PA, United States

**Keywords:** phenotypic covariation, developmental covariation, height-diameter allometry, functional mapping, QTL network, woody plant

## Abstract

How one trait developmentally varies as a function of others shapes a spectrum of biological phenomena. Despite its importance to trait dissection, the understanding of whether and how genes mediate such developmental covariation is poorly understood. We integrate developmental allometry equations into the functional mapping framework to map specific QTLs that govern the correlated development of different traits. Based on evolutionary game theory, we assemble and contextualize these QTLs into an intricate but organized network coded by bidirectional, signed, and weighted QTL-QTL interactions. We use this approach to map shoot height-diameter allometry QTLs in an ornamental woody species, mei (*Prunus mume*). We detect “pioneering” QTLs (piQTLs) and “maintaining” QTLs (miQTLs) that determine how shoot height varies with diameter and how shoot diameter varies with height, respectively. The QTL networks inferred can visualize how each piQTL regulates others to promote height growth at a cost of diameter growth, how miQTL regulates others to benefit radial growth at a cost of height growth, and how piQTLs and miQTLs regulate each other to form a pleiotropic web of primary and secondary growth in trees. Our approach provides a unique gateway to explore the genetic architecture of developmental covariation, a widespread phenomenon in nature.

## Introduction

Understanding the pattern of how different traits interact with each other during developmental processes can gain insight into the mechanistic basis underlying plant productivity and adaptation to changing environments (Walker et al., 2017; Rosado et al., 2018). For example, the stemwood production of trees is determined by stem height and stem diameter that covary during ontogeny (Feldpausch et al., 2011). The allocation pattern of stem growth to height and diameter determines reflects a tree’s capacity to survive, reproduce, and adapt to environmental conditions (King et al., 2006; Hulshof et al., 2015; Bourque et al., 2019). The relationship between tree height and diameter varies substantially along spatial and environmental gradients. Trees that are more densely packed tend to have a higher height-diameter ratio than those that are more widely spaced. These differences may be associated with increased competition for sunlight in dense stands and increased capacity to resist wind stress in open-grown stands (Henry and Aarssen, 1999). Temperature and to some extent precipitation can in part explain the allometric allocation of stem height and diameter growth (Hulshof et al., 2015).

Trait covariation is determined by developmental processes where genetic and environmental factors intervene jointly. A number of studies suggest that the capacity by which plants produce coordinated variation among different traits, known as morphological integration (Olson and Miller, 1958; Hallgrímsson et al., 2009), is under genetic control (Andersson et al., 2007; Niklas and Marler, 2007; Kroon et al., 2008). For example, specific quantitative trait loci (QTLs) responsible for height-diameter covariation have been detected in a variety of tree species (Wu, 1998; Tsarouhas et al., 2002; Sun et al., 2014; Curtolo et al., 2017. Several studies have develop statistical approaches for mapping allometry quantitative trait loci (QTLs) that govern the developmental change of one trait as a function of another trait (Li et al., 2007; Huang et al., 2014). Stimulated by the importance of stem height-diameter allometry in tree genetics, we have extended these approaches to map different batteries of QTLs responsible for reciprocal changes of one trait with the other (Jiang et al., 2016). By analyzing genetic mapping data of stem height and diameter growth in *Populus* trees, our approach can map and detect the “pioneering” QTLs (piQTLs) that determine how height scales with diameter and “maintaining” QTLs (miQTLs) that determine how diameter scales with height. Interestingly, most of these QTLs have been validated through a detailed gene enrichment analysis, thereby thought to provide new insight into the genetic architecture of the developmental allometry between primary and secondary growth in woody plants.

While existing reductionist-based approaches aimed at characterizing individual significant QTLs are powerful for genetic dissection, it has become increasingly clear that complex traits, especially morphological integration between different but developmentally coordinated traits, may also be controlled by QTL-QTL interactions that coalesce into a highly intricate but coordinated network. A wealth of literature supporting network thinking has arisen from medical research (Barabási et al., 2011; Chan and Loscalzo, 2012), but in recent years a consensus has been reached on the necessity of using holistic, system-oriented approaches to study plant complex traits (Ogura and Busch, 2016; Lavarenne et al., 2018. Approaches for inferring various regulatory networks from genomic, proteomic, and transcriptomic data have been well developed and widely used as a routine approach for modern biological research (Mizrachi et al., 2017). However, the characterization of QTL interaction networks remains largely unexplored, mainly because no powerful statistical methods have been developed.

We argue that a QTL interacts with others through a rule that can be explained by game theory. Game theory, originated in economic research (von Neumann and Morgenstern, 1944), has been widely used as a tool to unravel some uncertain machineries behind social or biological complexities (Falster and Westoby, 2003). The integration of game theory and evolutionary biology has created a new discipline—evolutionary game theory (Smith and Price, 1973). Through mathematical modeling, this discipline has found its new applications to studying the developmental pattern of trait covariation (Zhu et al., 2016; Wang et al., 2017; Fu et al., 2018). The combination of functional mapping and evolutionary game theory has led to the identification of QTL-QTL networks for phenotypic plasticity (Ye et al., 2019). In this article, we extend this combination approach to reconstructing genetic networks that code how different QTLs interact with each other to affect developmental covariation. From such networks, we further identify key QTLs or key pathways that are the determinants of developmental allometry. To test and validate our framework, we initiated an experiment of genetic mapping for a well-studied woody plant species—mei (*Prunus mume*) (Sun et al., 2017; Zhang et al., 2018). We detected several piQTLs and miQTLs and coalesced these QTLs into the interacting networks for a better understanding of the genetic architecture underlying H-D allometry.

## Materials and Methods

### Functional Mapping of Developmental Covariation

As a quantitative description of morphological integration, trait covariation is defined as the coordinated change of one trait as a function of other traits. Developmental covariation describes how one trait changes as a function of other traits during ontogeny. We start our model derivations by considering the developmental covariation between stem height growth and stem diameter growth as an example. In our previous study, we also used this example to map height-diameter allometry QTLs (Jiang et al., 2016). We recapitulate part of our previous procedure as a first step for our QTL network inference.

A number of biologically meaningful mathematical equations have been developed to quantify the allometric relationship of stem height and diameter in trees (Henry and Aarssen, 1999; Hulshof et al., 2015). Let *H*(*t*) and *D*(*t*) denote stem height and diameter growth at age *t* (*t* = 1, …, *T*), respectively. One such representative model (Huang et al., 1992) is expressed as

(1A) $$H\left(t\right)=H_{0}+e^{a_{H\leftarrow D}+\frac{d_{H\leftarrow D}}{b_{H\leftarrow D}{+}^{D\left(t\right)}}}$$

(1B) $$D\left(t\right)=\frac{d_{D\leftarrow H}}{a_{D\leftarrow H}-\ln\left(H\left(t\right)-H_{0}\right)}-b_{D\leftarrow H}\;$$

where *H*_0_ is the stem height at an initiate time point at which stem height is measured; *a_H←D_*, *b_H←D_*, and *d_H←D_* are three power coefficients of stem height scaling with stem diameter; and *a_D←H_*, *b_D←H_*, and *d_D←H_* are those of stem diameter scaling with height. Although equations (1A) and (1B) are mathematically interchangeable in terms of describing the H-D relationship, they differ in the direction of allometric scaling. Equation (1A) describes how stem height increases in response to the increase in stem diameter through parameters *a_H←D_*, *b_H←D_* and *d_H←D_*, whereas equation (1B) specifies the impact of stem height on radial growth during ontogeny through parameters *a_D←H_*, *b_D←H_*, and *d_D←H_*. As will be shown below, (*a_H←D_*, *b_H←D_*, *d_H←D_*) and (*a_D←H_*, *b_D←H_*, *d_D←H_*) are two different sets of parameters, if the genotypic value of a QTL as a response at the left side of equation (1A) or (1B) is predicted by the phenotypic value as a dependent variable at the right side. Parameters *a_H←D_* and *a_D←H_* play an important role in mediating H-D allometry when independent variables grows bigger in a late stage, while *b_H←D_* and *b_D←H_* play the same role when independent variables are smaller in an early stage. Parameters *d_H←D_* and *d_D←H_* are the relative growth rates which determine the spread of the curve alongside the induced variable axis.

We integrated H-D allometry equations into functional mapping through a multiplicative likelihood (Jiang et al., 2016). Let *y_i_* = (*y_i_* (1), …, *y_i_* (*T*)) and *z_i_* = (*z_i_* (1), …, *z_i_* (*T*)) denote stem height and diameter growth for individual *i* of a mapping population measured at a series of *T* time points, respectively. Their likelihoods are formulated as

(2A) $$L\left(y\right)=\overset{J}{\underset{j=1}{\prod }}\overset{n_{j}}{\underset{i=1}{\prod }}f_{j}^{H}\left(y_{i};\mu_{j|i}^{H},\Sigma_{i}^{H}\right)$$

(2B) $$L\left(z\right)=\overset{J}{\underset{j=1}{\prod }}\overset{n_{j}}{\underset{i=1}{\prod }}f_{j}^{D}\left(z_{i};\mu_{j|i}^{D},\Sigma_{i}^{D}\right)\;$$

where *j* stands for the *j*th genotype of a SNP (*j* = 1,…, *J*); *n_j_* is the observation of the *j*th genotype; $f_{j}^{H}\left(y_{i};\mu_{j|i}^{H},\Sigma_{i}^{H}\right)$ and $f_{j}^{D}\left(z_{i};\mu_{j|i}^{D},\Sigma_{i}^{D}\right)$ are multivariate normal distributions of stem height and diameter, respectively, with expected mean vector of genotype *j* for sample *i* over *T* time points, expressed as

(3A) $$\mu_{j|i}^{H}=\left(\mu_{j|i}^{H}\left(1\right),\mu_{j|i}^{H}\left(2\right),\;\;\ldots \;\;\ldots \;\;,\;\;\mu_{j|i}^{H}\left(T\right)\right)$$

(3B) $$\mu_{j|i}^{D}=\left(\mu_{j|i}^{D}\left(1\right),\mu_{j|i}^{D}\left(2\right),\;\;\ldots \;\;\ldots \;\;,\;\mu_{j|i}^{D}\left(T\right)\right)$$

and with a (*T*×*T*) longitudinal matrix composed of time-dependent variances and covariances expressed as

(4A) $$\Sigma_{i}^{H}=\left[\begin{array}{ccc} \sigma_{H}^{2}\left(1\right) & \cdots & \sigma_{H\left(1,T\right)}\\ \vdots & \ddots & \vdots \\ \sigma_{H\left(T,1\right)} & \cdots & \sigma_{H}^{2}\left(T\right) \end{array}\right]$$

(4B) $$\Sigma_{i}^{D}=\left[\begin{array}{ccc} \sigma_{D}^{2}\left(1\right) & \cdots & \sigma_{D\left(1,T\right)}\\ \vdots & \ddots & \vdots \\ \sigma_{D\left(T,1\right)} & \cdots & \sigma_{D}^{2}\left(T\right) \end{array}\right]$$

We modeled the structure of genotype-specific mean vector (2A) and (2B) by allometry growth equations (1A) amd (1B), which are expressed as

(5A) μj|iH=(μj|iH(1),  …  …  , μj|iH(T))μj|iH=(Hi0+eajH←D+djH←DbjH←D+i(z1),   …  , Hi0+eajH←D+djH←DbjH←D+i(zT))

(5B) μj|iD=(μj|iD(1),  …  …  , μj|iD(T))μj|iD=(djD←HajD←H−ln⁡(yi(1)−H0)−bjD←H, … , djD←HajD←H−ln⁡(yi(T)−H0)−bjD←H)

where $\mu_{j|i}^{H}(t)$ and $\mu_{j|i}^{D}(t)$ are the genotypic value of stem height and diameter at age *t*, respectively; and power parameters (*a_jH←D_*, *b_jH←D_*, *d_jH←D_*) and (*a_jD←H_*, *b_jD←H_*, *d_jD←H_*) are defined for genotype *j*. Equations (5A) and (5B) suggest that the genotypic value of a growth trait at a time is modelled by the phenotypic value of its allometrically related trait.

Functional mapping models the covariance matrices (4A) and (4B) by a parsimonious and flexible approach, such as an autoregressive (Ma et al., 2002), antedependence (Zhao et al., 2005), autoregressive moving average (Li et al., 2010), or nonparametric and semiparametric approaches (Das et al., 2011). The first-order autoregressive [AR(1)] approach is computationally efficient, but needs the stationarity assumption. Compared with this approach, the others are more flexible, but may be computationally more expensive. Functional mapping has been implemented with several statistical algorithms for obtaining the maximum likelihood estimates (MLEs) of genotype-specific growth parameters and the parameters that model the covariance structure. These algorithms include the EM algorithm and some optimization techniques, like the simplex algorithm (Ma et al., 2002; Zhao et al., 2005).

### Testing Pioneering QTLs or Maintaining QTLs and Estimating Their Temporal Genetic Effects

To test whether a particular SNP is associated with height–diameter growth allometry, we just need to test whether a set of power parameters (*a_jH←D_*, *b_jH←D_*, *d_jH←D_*), or (*a_jD←H_*, *b_jD←H_*, *d_jD←H_*) differs jointly among genotypes. This can be done, respectively, by formulating the following two sets of hypotheses:

(6A) $$\begin{array}{l} H_{0}:\left(a_{jH\leftarrow D},\;b_{jH\leftarrow D},\;d_{jH\leftarrow D}\right)\\ \;\equiv \left(a_{H\leftarrow D},\;b_{H\leftarrow D},\;d_{H\leftarrow D}\right),\;for\;all\;j=1,\;\ldots ,J\\ \;\;\;\;\;\;\;\;\;\;H_{1}:\;\text{At least one of the equalities in the}\;H_{0}\;\text{does not hold} \end{array}$$

(6B) $$\begin{array}{l} H_{0}:\left(a_{jD\leftarrow H},\;b_{jD\leftarrow H},\;d_{jD\leftarrow H}\right)\\ \;\equiv \left(a_{D\leftarrow H},\;b_{D\leftarrow H},\;d_{D\leftarrow H}\right),\;for\;all\;j=1,\;\ldots ,J\\ \;\;\;\;\;\;\;\;\;\;H_{1}:\;\text{At least one of the equalities in the}\;H_{0}\;\text{does not hold} \end{array}$$

After the MLEs of the unknown parameters under the *H*_0_ and *H*_1_ for each test are obtained, the log-likelihood ratio (LR) is calculated. By comparing it with the critical threshold of a chi-square distribution, the P-value reflecting the significance of the SNP considered is then obtained. The approach for adjusting for multiple comparisons, such as Bonferroni, to take into account all SNPs was used to obtain a genome-wide threshold. The SNPs whose significance level is beyond the adjusted criterion are regarded as QTLs. The significance tests based on hypotheses (6A) and (6B) produce ecologically different interpretations. Hypothesis (6A) intends to find a QTL that modulates how height growth increases per diameter growth, a process critically determining a tree’s capacity to pioneer growth space. Such a QTL is called the “pioneering” QTLs or piQTLs. On the other hand, hypothesis (6B) can detect the so-called “maintaining” QTLs or miQTLs that regulates the increase of stem growth per height growth, which is related to the capacity of a tree to maintain growth space.

For those QTLs tested to be significant based on hypothesis tests (6A) and (6B), we calculated their genetic effects or genetic variances over developmental allometry. For example, if a piQTL *k* is a testcross QTL with two genotypes *k_j_* (*j* = 1, 2), then its genetic effect on the allometry of height growth with diameter growth is calculated from genotypic values as shown in equations (5A), expressed as

(7A) $$P_{k}\left(t\right)=\mu_{k_{1}}^{H}\left(t\right)-\mu_{k_{2}}^{H}(t),\;with\;\mu_{k}^{H}\left(t\right)=\sum_{i=1}^{n_{k_{j}}}\mu_{k_{j}|i}^{H}\left(t\right)\;$$

Likewise, the genetic effect of a miQTL *k* on the allometry of diameter growth with height growth is calculated as

(7B) $$M_{k}\left(t\right)=\mu_{k_{1}|i}^{D}\left(t\right)-\mu_{k_{2}|i}^{D}(t),\;with\;\mu_{k|i}^{D}\left(t\right)=\sum_{i=1}^{n_{k_{j}}}\mu_{k_{j}|i}^{D}\left(t\right)$$

Note that the genetic effects of QTLs estimated from equations (7A) and (7B) are those that temporally change.

### Reconstructing Genetic Networks of QTL-QTL Interactions

After the genetic effects of all QTLs are estimated, we formulate an evolutionary game theory model that specifies how one QTL is expressed depending on its own intrinsic property and the regulation of other QTLs on it. For a set of *p* piQTLs or *m* miQTLs, this can be mathematically expressed as a system of ordinary differential equations (ODEs), respectively,

(8A) $$\frac{dP_{k}\left(t\right)}{dt}=U_{k}\left(P_{k}\left(t\right):\Phi_{k}\right)+\overset{p}{\underset{k^{\prime }=1,k^{\prime }\neq k}{\sum }}U_{kk^{\prime }}\left(P_{k^{\prime }}\left(t\right):\Phi_{kk^{\prime }}\right),\;k\;=\;1,\;\;\ldots \;,\;p$$

(8B) $$\frac{dM_{k}\left(t\right)}{dt}=V_{k}\left(M_{k}\left(t\right):\Psi_{k}\right)\;+\overset{m}{\underset{k^{\prime }=1,k^{\prime }\neq k}{\sum }}V_{kk^{\prime }}\left(M_{k^{\prime }}\left(t\right):\Psi_{kk^{\prime }}\right),\;k\;=\;1,\;\;\ldots \;,\;m$$

where the change of QTL *k*’s genetic effect per unit time is split into two component: the first describes the main effect of this QTL that is expressed independently from the effects of any other QTLs, specified by a QTL-specific smoothing function *U_k_*(*P_k_*(*t*):Φ_k_) for piQTLs or *V_k_*(*M_k_*(*t*):Ψ_k_) for miQTLs; and the second reflects an aggregated effect of the influences of all other SNPs on QTL *k*, specified by the sum of smoothing functions $\sum_{k^{\prime }=1,k^{\prime }\neq k}^{p}U_{kk^{\prime }}\left(P_{k^{\prime }}\left(t\right):\Phi_{kk^{\prime }}\right)$ for piQTLs or $\sum_{k^{\prime }=1,k^{\prime }\neq k}^{m}V_{kk^{\prime }}\left(M_{k^{\prime }}\left(t\right):\Psi_{kk^{\prime }}\right)$ for miQTLs. By estimating a set of ODE parameters Φ*_k_* or Ψ*_k_*, we can determine the pattern and magnitude of the independent effect of individual QTLs. Similarly, the estimation of another set of ODE parameters Φ*_kk_* or Ψ*_kk_* enables us to characterize whether and how the effect of a QTL *k* depends jointly on all other QTLs. The forms of smoothing functions can be derived parametrically or non-parametrically, although a nonparametric approach can provide a general formulation [38].

### Biological Interpretation of QTL-QTL Interactions

The strategy with which a QTL chooses to regulate or repress other QTLs based on the latter’s strategy can be interpreted by game theory. Although game theory is based on the rationality assumption, evolutionary game theory models the strategy of a QTL that interacts with others in either rational or irrational way. The quantitation of evolutionary game theory through a system of ODEs (8A and 8B) makes the dissection of the overall genetic effect of a focal QTL into its independent component and its dependent component affected by all possible other QTLs. We show that the fundamental principle of community ecology can be used to explain the dependent component.

In general, a QTL regulates others through the strategy of activation (+), neutrality (0), or repression (–). Consider two QTLs A and B, whose mutual strategies can be described in a matrix form:

(9) $$\begin{array}{cc} \; & \begin{array}{ccc} \; & \text{QTL B} & \;\\ + & 0 & - \end{array}\\ \text{QTL A} & \begin{array}{c} \begin{array}{c} +\\ \begin{array}{c} 0\\ - \end{array} \end{array}\left(\begin{array}{ccc} +/+ & +/0 & +/-\\ 0/+ & 0/0 & 0/-\\ -/+ & -/0 & -/- \end{array}\right) \end{array} \end{array}$$

As shown in Ye et al. (2019), we classify QTL interactions into the following types: *symmetric positive epistasis* (+/+)—two QTLs activate each other; *symbiosis* (0/0)—two QTLs do not affect each other; *negative epistasis* (–/–)—QTLs repress each other; *directional positive epistasis* (+/0 or 0/+)—one QTL activates its partner whereas the latter does not affect the former; *directional negative epistasis* (0/– or –/0), one QTL represses the other but the latter does not influence the former; and *altruistic or repressive epistasis* (+/– or –/+)—one QTL activates the other but is repressed by the latter. These patterns of regulation contain the direction and sign QTL-QTL interactions. The estimates of the dependent components from equations (8A) and (8B) will not only provide the direction and sign of genetic interactions but also the strengthen of each interaction.

### Experimental Design

To test and validate our model for illustrating the QTL network of stem H-D allometry, we designed and conducted an experiment of QTL mapping from full-sib families of mei, a woody plant. Mei, native to China, is an ornamental species of great economic value. Its colorful flowers, pleasant fragrance, and cold-hardiness make it a widely cultivated plant (Chen, 1996). Because of its genome sequencing (Zhang et al., 2011), mei has been widely used as a model system to study the genetic architecture of complex traits in woody plants and the evolution of *Prunus* species (Sun et al., 2014; Sun et al., 2017; Zhang et al., 2018).

The ornamental value of mei mainly lies in its flowers, with an incredible diversity of floral size, color, shape, flower number, and flowering phenology (Zhang et al., 2018). However, all these floral traits are affected by the botanical structure of mei trees (Sun et al., 2017). The allometrical relationship of stem height and diameter growth is a determinant of mei’s branch display, leaf area, and growth vigor, and an understanding of its genetic control can help mei breeders select superior varieties of desirable ornamental features.

We established three full-sib families as mapping populations by crossing three pairs of varieties: F-2014 of 156 progeny derived from Fenban (female) and Kouzi Yudie (male), Y-2015 of 184 progeny derived from Liu Bandan (female) and Sanlun Yudie (male), and L-2015 of 190 progeny derived from Liuban (female) and Huang Lve (male). Scions from the seedlings of these three families were grafted on 5-year rootstocks of healthy mei trees in a winter time at the Experimental Station of Beijing Forestry University Center for Computational Biology, located in Nantong, Jiangsu Province, southeast China. In the coming spring, scions sprout into shoots. Ten randomly chosen shoots from each progeny were measured for their heights and diameters at base once every two weeks, starting at 1 week since sprouting and ending when trees stop their growth in the fall. To investigate yearly variation in shoot growth, trait phenotyping was repeated for family F-2014 in 2015.

DNA samples extracted from young leaves were used for SNP genotyping. After SNP calling, we extracted the SNPs with overall sequencing depths of more than 8, quality scores over 30, and at least four uniquely mapped reads per allele (Sun et al., 2013). The three families were genotyped for 1,484 segregating SNPs (261 testcross markers and 1,223 intercross markers) in the F-2014 population, 5,393 segregating SNPs (3,986 testcross markers and 1,407 intercross markers) in the Y-2015 population, and 5,012 segregating SNPs (4,477 testcross markers and 535 intercross markers) in the L-2015 population. A testcross marker is one at which one parent is heterozygous but the other is homozygous, whereas an intercross marker is one at which both parents are heterozygous (Maliepaard et al., 1997; Wu et al., 2002; Lu et al., 2004; Tong et al., 2011).

## Results

### Identification of piQTLs and miQTLs

**Figure 1:**
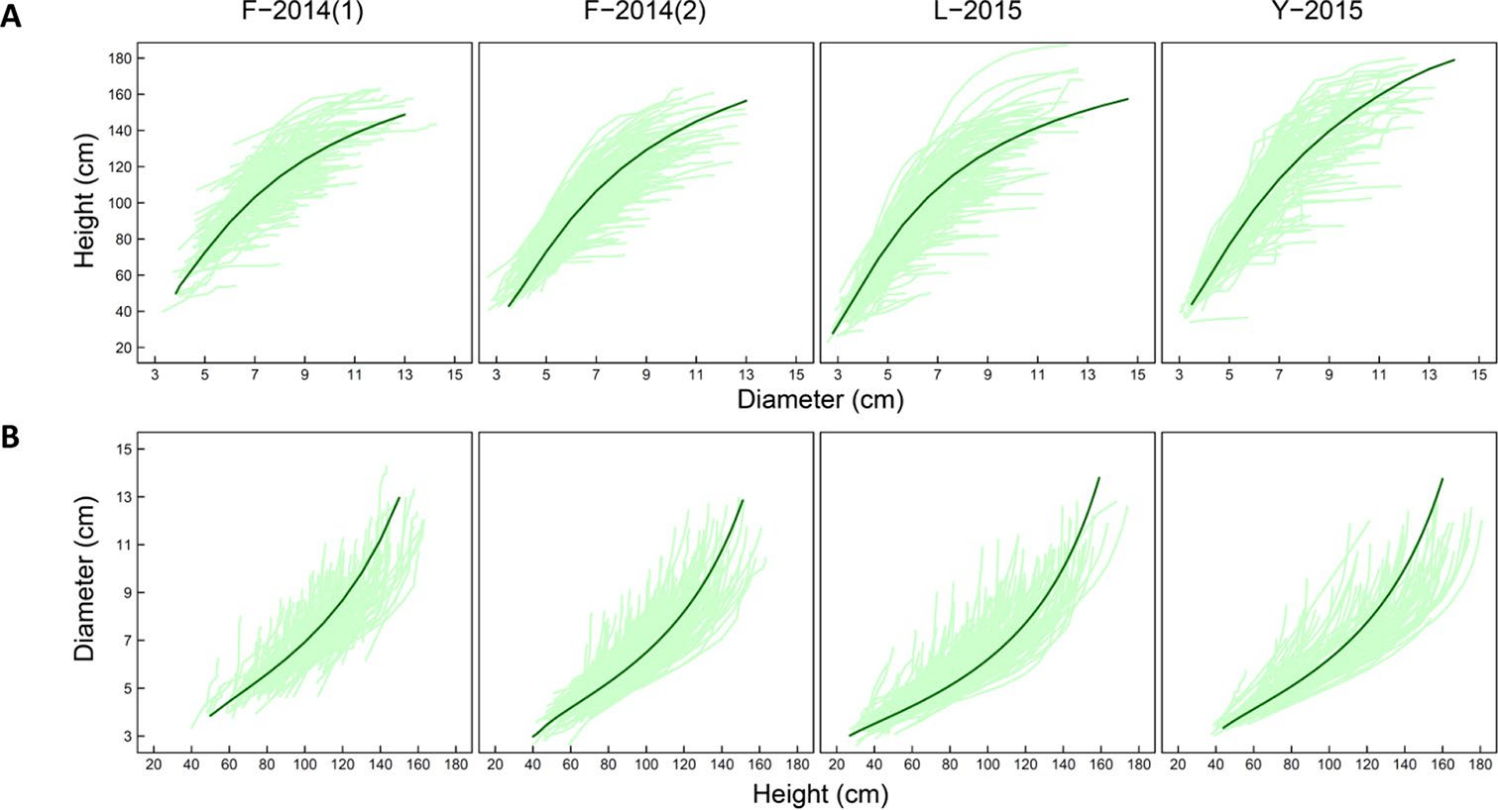
Developmental covariation showing how shoot height varies as a function of shoot diameter **(A)** and how shoot diameter varies as a function of shoot height **(B)** during ontogeny in different mapping materials, F-2014 measured in 2 years, L-2015, and Y-2015. Light green lines represent height-diameter developmental allometry curves of each progeny and a dark green line is the mean allometry curve of all progeny fitted by Equation (1A) and (1B), respectively.

We illustrate the allometric trajectories of stem height against stem diameter (**Figure 1A**) and the allometric trajectories of stem diameter against stem height (**Figure 1B**) for families F-2014 (in 2 years), Y-2014 (in 1 year), and L-2015 (in 1 year), totalizing four research materials. Huang et al. (1992) assessed a list of commonly used H-D relationship equations. By fitting each of these equations to the mean curves of all progeny from each dataset, we found that equations (1A) and (1B) are the most parsimonious for curve fitting based on AIC values calculated. Functional mapping implemented with equations (1A) and (1B) was used to map piQTLs and miQTLs. It appears that variation in H-D allometry among progeny in all materials is quite stationary over dependent variables. Thus, the structure of residual covariance matrices (4A) and (4B) was modelled by the computationally efficient AR(1) approach.

**Figure 2:**
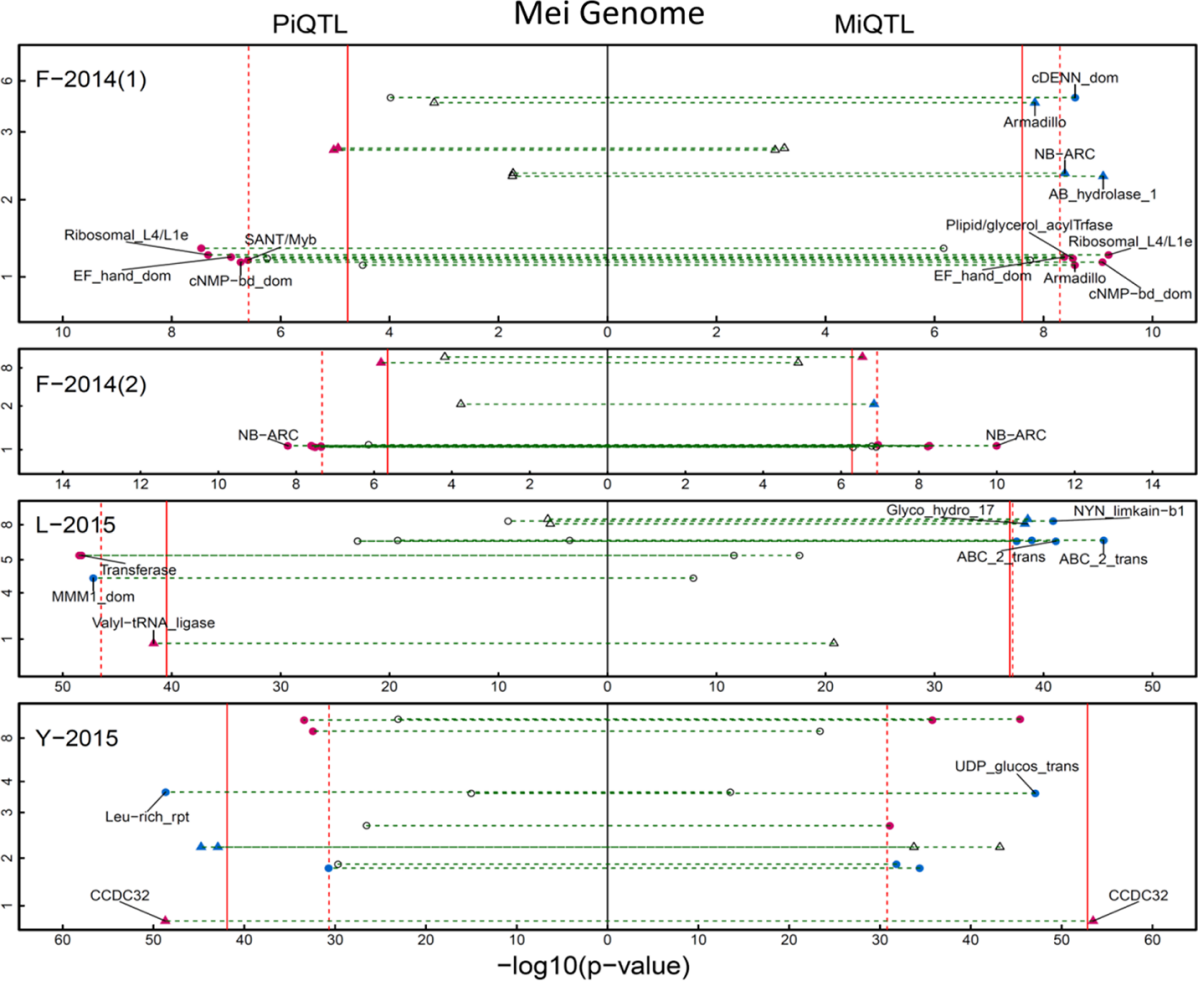
Manhattan plots of “pioneering” QTL (piQTL) (left) and “maintaining” QTL (miQTL) detection (right) on the mei genome in different mapping materials, F-2014 measured in 2 years, L-2015, and Y-2015. X-axis represents –log(P) value and y-axis represents mei’s eight chromosomes. Triangles and dots stand for intercross QTLs and testcross QTLs, respectively, with solid and dash lines denoted as the genome-wide thresholds of intercross QTL and testcross QTL detection at the 5% significance level. As comparison, the significance level at which a piQTL affects how diameter varies with height and a miQTL affects how diameter varies with height is also indicated. Names of the genes that reside in the QTL region are shown.

We detected a number of significant QTLs distributed on various chromosomal positions that regulate H-D allometry in three mapping populations (**Figure 2**). Family F-2014 contains seven piQTLs and 9 miQTLs in year 1 and seven piQTLs and six miQTLs in year 2. SNPs 101832, 104243, and 105979 were identified simultaneously as piQTLs and miQTLs in year 1, and 101200 and 99391 identified as piQTLs and miQTLs in year 2. We did not detect any pleiotropic piQTLs that affect how shoot height growth scales with shoot diameter growth across years and any miQTLs that affect how shoot diameter growth scale with shoot height across years. We detected seven piQTLs and seven miQTLs in family Y-2015 and seven piQTLs and four miQTLs in family L-2015, and in family Y-2015, a QTL (SNP 6,355) behaves as both piQTLs and miQTLs. The roles played by piQTLs and miQTLs differ in terms of their function: piQTLs modulate the ecological process of how stem diameter invests stem height to facilitate trees to capture spatial advantage for optimal fitness in a competitive environment, whereas miQTLs control a different ecological process in which spatial advantage can be maintained by investing radial growth.

Next, we performed a GO analysis to interpret the biological functions of each piQTL and each miQTL detected. Most piQTLs residue in the chromosomal regions of many candidate genes encoding cell metabolism, growth and differentiation (**Figure 2**; **Table S1**). All these functions can be linked to stem growth. For example, piQTL 70.833/19860026 is in the proximity of genes that encode Leucine-rich repeat playing an important role in division and differentiation of stem cells and growth of apical meristem. piQTL 0.808/330310 situates in the gene family linked to plant peroxidase that, as a key enzyme, participates in lignin deposition. Among 32 miQTLs detected in F-2014 population, 31 are related to candidate genes of known function (**Table S1**). The biological function of those QTLs whose annotations are not available remains to be validated.

**Figure 3:**
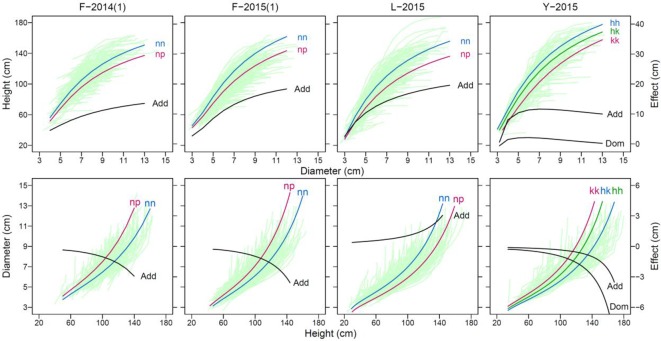
Genotype (nn and np)-dependent curves of developmental covariation between shoot height and shoot diameter at a significant “pioneering” QTL (piQTL) and “maintaining” QTL (miQTL) detected in different mapping materials, F-2014 measured in 2 years, L-2015, and Y-2015. The genetic effect curve of each QTL is also shown, where “Add” denotes the additive effect calculated as the homozygote—the heterozygote and “Dom” denotes the dominant effect calculated as the heterozygote—half of the sum of the two homozygotes.

Functional mapping can illustrate how genetic effects of QTLs change developmentally over time course. To demonstrate the temporal change of QTL effects, we chose a significant piQTL and miQTL for each material to draw genotypic curves (**Figure 3**). We found that the genetic effect of each QTL increases with diameter growth for piQTLs and with height growth for miQTLs in all materials, consistent between 2 years for F-2014. However, the slope of effect increase is more abrupt for miQTLs than for piQTLs for all materials.

### QTL Control Networks

**Figure 4:**
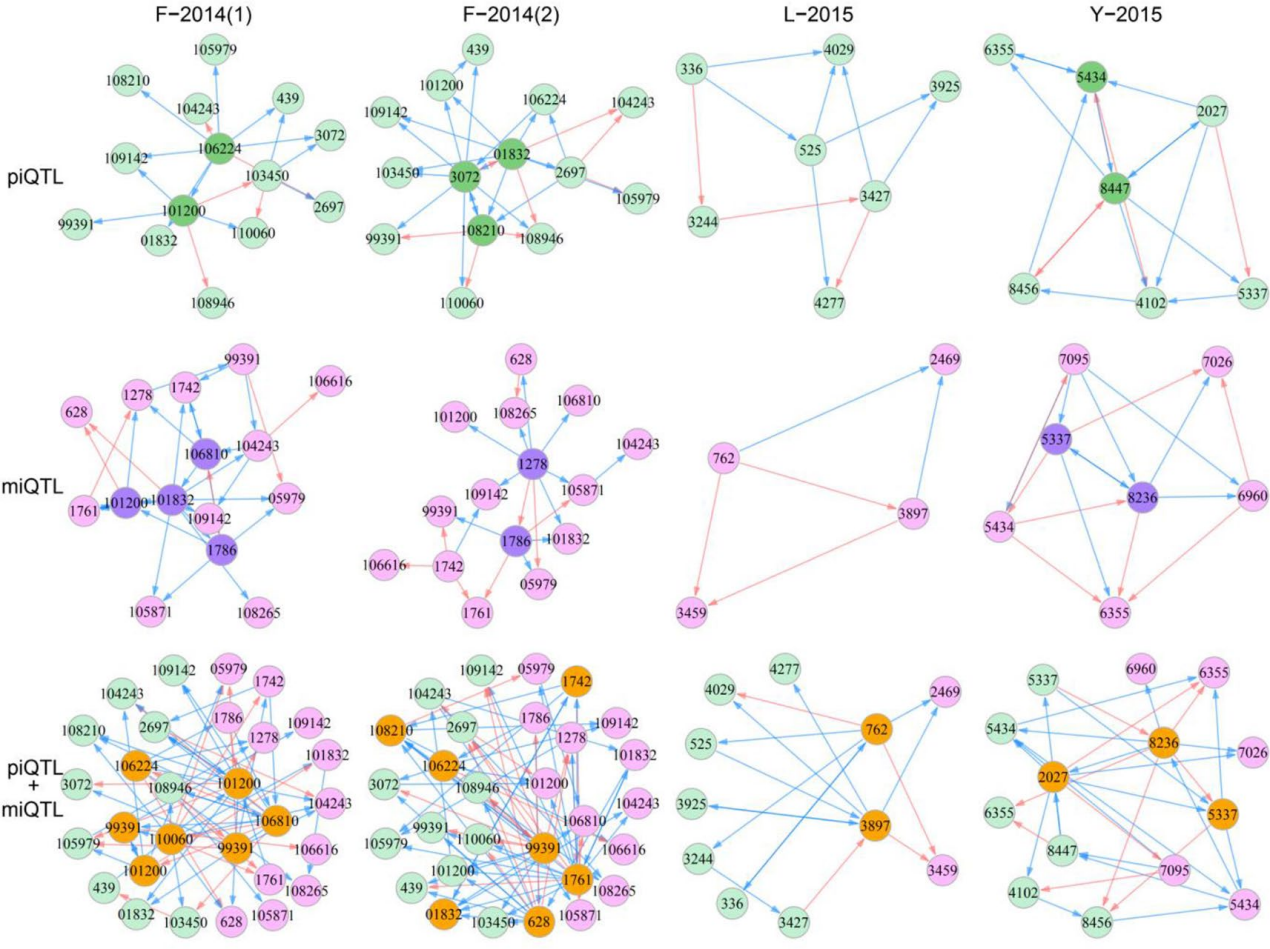
QTL control networks that govern developmental covariation between shoot height and shoot diameter in different mapping materials, F-2014 measured in two years, L-2015, and Y-2015. The networks were reconstructed by “pioneering” QTLs (piQTLs), “maintaining” QTLs (miQTLs), and piQTLs + miQTLs, respectively, where dark circles denote hub QTLs, red arrowed lines stand for promotion, and blue arrowed lines stand for inhibition.

Based on allometry-dependent genetic effects of each QTL, we reconstructed the control networks of piQTLs and miQTLs for the four materials, respectively (**Figure 4**). A piQTL control network describes how different piQTLs interact with each other to control the allometric change of stem height growth with stem diameter growth. Similarly, a miQTL control network characterizes the impact of a web of miQTL-miQTL epistasis on the allometric change of stem diameter growth with stem height growth. We also reconstructed a joint QTL control network among piQTLs and miQTLs to illustrate how these two types of QTLs interact with determine H-D allometry. Three types of networks constituted by piQTLs, miQTLs, and a mix of piQTLs and miQTLs are sparse, suggesting that a single QTL may only interact with a limited number of others. However, a few QTLs within each network act as hubs, playing a central role in network behavior through linking more QTLs than the average. We found that directional positive epistasis and directional negative epistasis are two major links that constitute QTL control networks.

For F-2014(1), piQTLs 106224 and 101200 are two hubs that each regulate many other QTLs, although 101200 is regulated by 106224. We found that both hub QTLs trigger directional negative epistasis with most of these regulated QTLs because the latter has no effect on the former. QTL 101200 is also a hub in the miQTL network, but it receives many incoming regulations from other miQTLs. piQTL 99391 is a peripheral QTL because it resides at the edge of the piQTL network, with a single link with 101200. However, it is interesting to find that 99391 is a hub in the joint piQTL and miQTL network that links with many miQTLs. QTL 106810 is a hub within the miQTL network, and it is still a hub in the joint piQTL and miQTL network, but it also interacts with many piQTLs. 110060 is a piQTL, but it links with both piQTLs and miQTLs in the joint network. Taken together, as two highly coordinated processes, how stem height growth scales with stem diameter growth and how stem diameter growth scales with stem height growth are controlled not only directly by pleiotropic QTLs, such as 101200 and 99391 but also indirectly through the links of piQTLs and miQTLs. For L-2015 and Y-2015 materials, we have also identify similar features of piQTL, miQTL, and piQTL-miQTL networks (**Figure 4**). Overall, all QTL networks are dominated by directional negative and positive epistasis, including a few cases of symmetric positive and negative epistasis.

QTL networks display topological changes with year (**Figure 4**). For example, hubs of the piQTL network are 106224 and 101200 in the first year, but they become 3072, 01832, and 108210 in the second year. Although 439 and 105979 are peripheral QTLs in both years, the QTLs with which they link vary with year. miQTL 101200 is a hub that links with many QTLs in the first year, but it becomes a periphery in the second year. There are many other QTLs that determine year-year variation in the developmental allometry of stem diameter with stem height. We can also identify year-dependent differences in the topological structure of the piQTL-miQTL network. Taken together, H-D allometry undergoes a year-specific change, which may be driven by QTL networks.

### Computer Simulation

**Figure 5:**
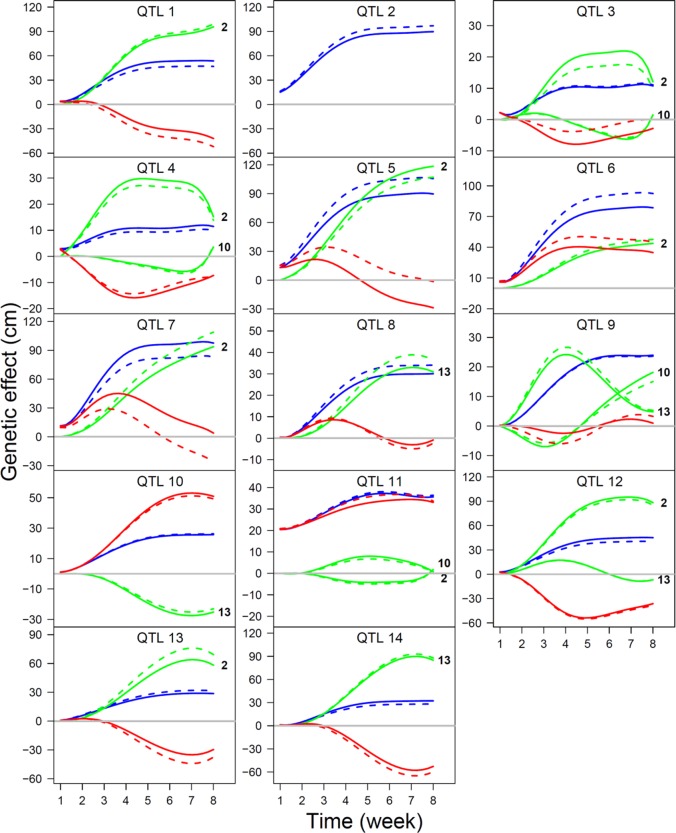
The estimates of time-varying genetic effect curves (slash line) under simulation scenario *T* = 30 and *υ*^2^ = 0.5, in a comparison with true genetic effects curves (solid line), for a 14-QTL interaction network. The overall curve (blue) is decomposed into its underlying independent curve (red) and dependent curves (green). The QTLs that affect a focal QTL are shown at the end of curve.

We performed simulation studies to investigate the statistical properties of our QTL-networking model. We simulated two allometrically related growth traits by mimicking the allometric relationships of shoot length growth with shoot diameter growth in the F-2014 mapping population. We simulated the data of genetic effects on these two traits by assuming the involvement of 14 QTLs that interact with each other in a network characterized by

(10) $$\{\begin{array}{l} \frac{dQ_{1}\left(t\right)}{dt}=U_{1}\left(Q_{1}\left(t\right):\Phi_{1}\right)+U_{1,2}\left(Q_{2}\left(t\right):\Phi_{1,2}\right)\\ \;\;\;\;\;\;\;\;\;\;\;\;\;\;\;\;\;\;\;\;\;\;\;\;\;\frac{dQ_{2}\left(t\right)}{dt}=U_{2}\left(Q_{2}\left(t\right):\Phi_{2}\right)\\ \frac{dQ_{3}\left(t\right)}{dt}=U_{3}\left(Q_{3}\left(t\right):\Phi_{3}\right)+U_{3,2}\left(Q_{2}\left(t\right):\Phi_{2,3}\right)++U_{3,10}\left(Q_{10}\left(t\right):\Phi_{3,10}\right)\\ \frac{dQ_{4}\left(t\right)}{dt}=U_{4}\left(Q_{4}\left(t\right):\Phi_{4}\right)+U_{4,2}\left(Q_{2}\left(t\right):\Phi_{4,2}\right)++U_{4,10}\left(Q_{10}\left(t\right):\Phi_{4,10}\right)\\ \;\;\;\;\;\;\;\;\;\;\;\;\;\;\;\;\;\;\;\;\;\;\;\;\;\;\;\;\;\;\;\frac{dQ_{5}\left(t\right)}{dt}=U_{5}\left(Q_{5}\left(t\right):\Phi_{5}\right)+U_{5,2}\left(Q_{5}\left(t\right):\Phi_{5,2}\right)\\ \;\;\;\;\;\;\;\;\;\;\;\;\;\;\;\;\;\;\;\;\;\;\;\;\;\;\;\;\;\;\;\frac{dQ_{6}\left(t\right)}{dt}=U_{6}\left(Q_{6}\left(t\right):\Phi_{6}\right)+U_{6,2}\left(Q_{6}\left(t\right):\Phi_{6,2}\right)\\ \;\;\;\;\;\;\;\;\;\;\;\;\;\;\;\;\;\;\;\;\;\;\;\;\;\;\;\;\;\;\;\frac{dQ_{7}\left(t\right)}{dt}=U_{7}\left(Q_{7}\left(t\right):\Phi_{7}\right)+U_{7,2}\left(Q_{7}\left(t\right):\Phi_{7,2}\right)\\ \;\;\;\;\;\;\;\;\;\;\;\;\;\;\;\;\;\;\;\;\;\;\;\;\;\;\;\;\;\;\;\frac{dQ_{8}\left(t\right)}{dt}=U_{8}\left(Q_{8}\left(t\right):\Phi_{8}\right)+U_{8,13}\left(Q_{13}\left(t\right):\Phi_{8,13}\right)\\ \frac{dQ_{9}\left(t\right)}{dt}=U_{9}\left(Q_{9}\left(t\right):\Phi_{9}\right)+U_{9,10}\left(Q_{10}\left(t\right):\Phi_{9,10}\right)++U_{9,13}\left(Q_{13}\left(t\right):\Phi_{9,13}\right)\\ \;\;\;\;\;\;\;\;\;\;\;\;\;\;\;\;\;\;\;\;\;\;\;\;\frac{dQ_{10}\left(t\right)}{dt}=U_{10}\left(Q_{10}\left(t\right):\Phi_{10}\right)+U_{10,13}\left(Q_{13}\left(t\right):\Phi_{10,13}\right)\\ \frac{dQ_{11}\left(t\right)}{dt}=U_{11}\left(Q_{11}\left(t\right):\Phi_{11}\right)+U_{11,2}\left(Q_{2}\left(t\right):\Phi_{11,2}\right)++U_{11,10}\left(Q_{10}\left(t\right):\Phi_{11,10}\right)\\ \frac{dQ_{12}\left(t\right)}{dt}=U_{12}\left(Q_{12}\left(t\right):\Phi_{12}\right)+U_{12,2}\left(Q_{12}\left(t\right):\Phi_{12,2}\right)++U_{12,13}\left(Q_{13}\left(t\right):\Phi_{12,13}\right)\\ \;\;\;\;\;\;\;\;\;\;\;\;\;\;\;\;\;\;\;\;\;\;\;\;\;\;\;\;\;\;\;\;\;\;\frac{dQ_{13}\left(t\right)}{dt}=U_{1}\left(Q_{13}\left(t\right):\Phi_{13}\right)+U_{13,2}\left(Q_{2}\left(t\right):\Phi_{13,2}\right)\\ \;\;\;\;\;\;\;\;\;\;\;\;\;\;\;\;\;\;\;\;\;\;\;\;\;\;\;\;\;\;\;\;\;\;\frac{dQ_{14}\left(t\right)}{dt}=U_{1}\left(Q_{14}\left(t\right):\Phi_{14}\right)+U_{14,13}\left(Q_{13}\left(t\right):\Phi_{14,13}\right) \end{array}$$

where QTL 2 is regarded as a hub QTL, and Φ and Φ are the ODE parameters that determine independent and dependent genetic effect of a QTL, respectively, whose values are given by the estimates of the F-2014 population. The residuals of the simulated genetic effects are assumed to follow a multivariate normal distribution with mean 0 and covariance matrix structured by an AR(1) model. Based on the number of longitudinal measurements (*T*) and time-constant variance (*υ*^2^), we used four simulation scenarios, (1) *T* = 10, *υ*^2^ = 0.05, (2) *T* = 10, *υ*^2^ = 0.5, (3) *T* = 30, *υ*^2^ = 0.05, and (4) *T* = 30, *υ*^2^ = 0.5. We examined and compared the statistical behavior of ODE parameter estimates under each scenario. As an example, we illustrate the estimation result under scenario 4 (**Figure 5**). We found that our model can reasonably well estimate ODE parameters and, therefore, reconstruct QTL networks under these simulation scenarios. The precision and accuracy of parameter estimates increase with increasing measurement number and/or decreasing residuals.

We further assessed the statistical power of our new model by estimating true positives (TP), false positive (FP), true positive rate (TPR), false positive rate (FPR), and the area under curve (AUC) of receiver operating characteristic curve (**Table 1**). In general, FPR are quite low under all simulation scenarios, but the power of the model is very much scenario-dependent. We do not recommend the use of a small number of measurements when residuals are large. If residuals are unavoidably large, the number of measurements should increase as much as possible to reach a good power of QTL network reconstruction. In general, when *υ*^2^ = 0.5 and T = 30, we obtain a power of 0.88.

**Table 1:** Statistical evaluation of QTL network reconstruction under four simulation scenarios.

*ν*^2^	T	TP	FP	TPR	FPR	AUC
0.05	10	13.6 (1.82)	34.7 (5.18)	0.52 (0.06)	0.07 (0.003)	0.61 (0.03)
	30	16.4 (1.69)	10.8 (4.37)	0.88 (0.04)	0.01 (0.003)	0.92 (0.02)
0.5	10	7.8 (3.27)	50.5 (7.38)	0.19 (0.09)	0.11 (0.007)	0.52 (0.07)
	30	12.3 (2.38)	27.9 (5.47)	0.51 (0.06)	0.08 (0.005)	0.67 (0.06)

## Discussion

Despite a universal phenomenon of fundamental importance to plant biology (Barbeito-Andrés et al., 2015), the genetic control mechanisms of developmental covariation have been poorly understood. Surprisingly, there is still no rich body of literature that reports the development of statistical approaches for mapping developmental allometry, although our recent work shows the promise of using a statistical model to map allometry QTLs (Jiang et al., 2016). Our previous model was based on reductionist thinking and, therefore, it may be powerful for identifying significant QTLs that act individually or pairwise (with epistasis), but fails to chart a global picture of genetic control from a holistic and system-oriented perspective. In this article, we develop a computational model for characterizing how each and every QTL interacts with others to determine trait allometry. Our QTL control networks fill a gap in complex-trait mapping by unraveling the emergent properties of the complex interplay of multiple QTLs.

As an example, our QTL networking model was used to address a fundamental question in tree biology, i.e., what is the genetic basis for the developmental allometry of primary growth (shoot height) vs. secondary growth (shoot diameter)? The ecological process of how stem diameter invests stem height determines a tree’s capacity to capture spatial advantage in a competitive environment. Given the same growth of stem diameter, a taller height has a better capacity to increase the exposure of leaves to light, increase the shading of competitors and elevate reproductive or dispersal organs than a shorter height. We created “pioneering QTLs” (or piQTLs) to define the QTLs that control the change in height growth with diameter growth, given that they play a pioneering role in stimulating trees to capture spatial resources for the optimal fitness [19]. On the other hand, the ecological process of how stem height invests stem diameter can help a tree to maintain its spatial advantage. Among those trees of the same height, stout ones can preserve the potential of growth towards next stages of competition than slender ones. We coined “maintaining QTLs” (or miQTLs) to define the QTLs that determine the increasing amount of radial growth at a cost of height growth. The identification of piQTLs and miQTLs helps to interpret the mechanistic basis of H-D allometry in response to environmental change.

In genetic mapping studies of a woody plant—mei (*Prunus mume*), we identified a set of piQTLs and miQTLs that determine the allometric variation of shoot heights vs. shoot diameters for scions grafted on rootstocks. These QTLs exert their effects on H-D allometry not only individually but also through a complex interaction network. We reconstructed piQTL control networks for the scaling process of height with diameter and miQTL networks for the scaling process of diameter with height for mei. In each network, we identified several hub QTLs that play a dominant role in network behavior by linking many other members. Because of the existence of these hub QTLs, other QTLs can be associated with H-D allometry through guilt by association in a gene network. To better respond to environmental change, a shoot needs to decide whether to prioritize height growth at a cost of diameter or diameter growth at a cost of height. Although each process is driven by its own network, these two processes as a whole to govern mei growth involve a coordinated network constituted by both piQTLs and miQTLs. In such a joint network, we identified piQTLs that affect the allometry of diameter with height through linking with miQTLs, and miQTLs that affect they allometry of diameter with height through linking with piQTLs. Such observations were seen from three different mapping populations.

Our QTL networking model offers systematic insights into the genetic architecture of developmental allometry between different phenotypic traits and thus extends beyond reductionist approaches that aim to detect the impacts of individual QTLs based on the law of parsimony. The model shows its utility for reconstructing QTL interaction networks underlying developmental covariation between stem height and stem diameter in trees, but its application is largely beyond this scope (Barabási et al., 2011). More recently, the advance of high-throughput phenotyping techniques has led to increasing amounts of high-dimensional phenotypic data that can better characterize the morphological, anatomical, physiological, and developmental features of plants as a coordinated whole to adapt to environmental change. Our model, coped to these data, will enable the recovery of intricate and dynamic QTL networks from which to identify key interaction pathways that can be genetically rewired to engineer or modify plant phenotypes of interest. Taken together, the new model could generate results that help to design more efficient, systems biology-based breeding strategies.

## Data Availability Statement

Computer code and data are available https://github.com/LiboJiang/QTLNetwork, or under request directly from the corresponding author.

## Author Contributions

LJ derived the model, performed data analysis, and wrote computer code. HS and MS participated in data analysis. MS, CZ, YC, XKZ and XLZ collected the data. LS conceived of the project. LS, TC, and QZ supervised the study. LS, LJ and RW wrote the manuscript.

## Funding

This research is supported by the National Natural Science Foundation of China (No. 31870689), the Fundamental Research Funds for the Central Universities (NO. 2015ZCQ-SW-06), the National Natural Science Foundation for Young Scientists of China (NO. 31600536, NO. 31700576), and grant 201404102 from the State Administration of Forestry of China.

## Conflict of Interest

The authors declare that the research was conducted in the absence of any commercial or financial relationships that could be construed as a potential conflict of interest.
