# Corrigendum: A Computational Model for Inferring QTL Control Networks Underlying Developmental Covariation

**DOI:** 10.3389/fpls.2020.00045

**Published:** 2020-02-13

**Authors:** Libo Jiang, Hexin Shi, Mengmeng Sang, Chenfei Zheng, Yige Cao, Xuli Zhu, Xiaokang Zhuo, Tangren Cheng, Qixiang Zhang, Rongling Wu, Lidan Sun

**Affiliations:** ^1^Beijing Advanced Innovation Center for Tree Breeding by Molecular Design, Center for Computational Biology, College of Biological Sciences and Biotechnology, Beijing Forestry University, Beijing, China; ^2^Beijing Advanced Innovation Center for Tree Breeding by Molecular Design, Beijing Key Laboratory of Ornamental Plants Germplasm Innovation & Molecular Breeding, National Engineering Research Center for Floriculture, Beijing Laboratory of Urban and Rural Ecological Environment, College of Landscape Architecture, Beijing Forestry University, Beijing, China; ^3^Center for Statistical Genetics, The Pennsylvania State University, Hershey, PA, United States

**Keywords:** phenotypic covariation, developmental covariation, height-diameter allometry, functional mapping, QTL network, woody plant

In the published article, there was an error regarding the affiliation for Lidan Sun. Instead of affiliation 3, it should be 2.

In addition, there is an error in the order of the Funding statement. The correct statement appears below. The authors apologize for this error and state that this does not change the scientific conclusions of the article in any way. The original article has been updated.

## Funding

This research is supported by the National Natural Science Foundation of China (No. 31870689), the Fundamental Research Funds for the Central Universities (NO. 2015ZCQ-SW-06), the National Natural Science Foundation for Young Scientists of China (NO. 31600536, NO. 31700576), and grant 201404102 from the State Administration of Forestry of China.

